# Body mass index, waist circumference and cognition over time – the Doetinchem Cohort Study

**DOI:** 10.1002/alz70860_106035

**Published:** 2025-12-23

**Authors:** Femke te Hoonte, Liset M. Rietman, Kirsten E.J. Wesenhagen, Susan H.J. Picavet, Monique W.M. Verschuren

**Affiliations:** ^1^ National Institute for Public Health and the Environment, Bilthoven, Utrecht, Netherlands; ^2^ Julius Center for Health Sciences and Primary Care, Utrecht, Utrecht, Netherlands

## Abstract

**Background:**

Obesity is a risk factor for dementia and cognitive decline (*Lancet* Commission, 2024). Questions remain about the impact of the timing of obesity over the life course and the obesity metric (body mass index (BMI, kg/m^2^) or waist circumference (WC, cm)). We studied the association between BMI, WC, and (abdominal) obesity and the level and decline of cognition over time.

**Method:**

Data from 3,918 participants (52% women) from the Doetinchem Cohort Study (rounds 2‐7, baseline age 45‐70) was used. Participants were extensively phenotyped including BMI, WC, and cognitive function at baseline and during follow‐up rounds (at 5‐year intervals). (Abdominal) obesity was defined as BMI ≥30 kg/m^2^ or WC >102 cm (men) or >88 cm (women). Cognitive domain scores (global cognition, memory, flexibility, and processing speed) were standardized into z‐scores based on the baseline measurement. Linear mixed models were used to study the association between time‐dependent BMI, WC, and (abdominal) obesity and level of and decline in the four cognitive domains. The effect of different age categories (below or above 55 years old at baseline) was tested using an interaction term. All models were sex‐stratified and adjusted for socio‐demographic factors, lifestyle factors and depressive symptoms.

**Result:**

Both BMI and WC were statistically significantly associated with level of global cognition, memory, flexibility, and processing speed in men and women. Per unit higher BMI, the effect size (z‐score) ranged from ‐0.011 (men) and ‐0.013 (women) for global cognition to ‐0.017 flexibility (men and women). Per cm higher WC, the effect size (z‐score) ranged from ‐0.004 for processing speed (men and women) to ‐0.009 (men) and ‐0.008 (women) for memory. Only in men aged 55+ at baseline, abdominal obesity was statistically significantly associated with accelerated decline in global cognition (z‐score ‐0.014) and flexibility (z‐score ‐0.017).

**Conclusion:**

In this study, higher BMI and WC were associated with lower levels of all four cognitive domains in men and women. BMI and WC were not associated with accelerated cognitive decline, apart from WC that was associated with accelerated decline (global cognition and flexibility) in men aged 55+ at baseline.

